# Successful management of congenital bronchial web in an adolescent using bronchoscopic ablation: A case report and review of literature

**DOI:** 10.1016/j.rmcr.2022.101786

**Published:** 2022-12-09

**Authors:** Sara G. Hamad, Ahmed Abushahin, Hisham Abdulsattar, Kashif Waqas, Mutasim Abu-Hasan

**Affiliations:** a Pediatric Pulmonology, Sidra Medicine, Doha, Qatar; b Pulmonology Medicine, Hamad Medical Corporation, Doha, Qatar; c Biomedical Engineering, Sidra Medicine, Doha, Qatar

**Keywords:** Bronchial web, Central airway obstruction, Congenital bronchial web, Endoscopic argon plasma ablation, Interventional bronchoscopy

## Abstract

Airway webs are abnormal fibrous membranes in the airway lumen that rarely occur but can lead to serious or even life-threatening symptoms because of critical airway obstruction. Airway webs can be acquired or congenital. Acquired webs are likely to be secondary to trauma, infections, or neoplasm. Congenital laryngeal, subglottic and tracheal webs present early in infancy or childhood and are more common than congenital bronchial webs. To our knowledge, there are a few reports on the bronchial web in the literature, and the true incidence of these lesions is unknown as many probably go undetected across the lifespan.

We here report a case of a congenital bronchial web and provide a review of the literature of all reported bronchial webs. Our patient is a teenage boy who was diagnosed with a congenital bronchial web obstructing the right main-stem bronchus (RMB) and causing right lung hypoplasia and persistent right middle and right lower lobe collapse. The web was treated successfully using endoscopic ablation by argon plasma coagulation and balloon dilatation. Treatment resulted in remarkable relief of right main stem obstruction and significant improvement in right lung collapse as well as clinical, spirometric, and radiological findings.

Due to the rarity of bronchial web, the clinical knowledge and the bronchoscopic interventional strategies demonstrated of this report make it relevant. Furthermore, it emphasizes that early diagnosis and management lead to favorable clinical outcomes.

## Abbreviations

APC: Argon plasma coagulation
BPD: Bronchopulmonary Dysplasia
BW: Bronchial web
FB: Flexible Bronchoscopy
RLL: Right lower lobe
RMB: Right main-stem bronchus
RML: Right middle lobe
RPA: Right pulmonary artery

## 1 Case

The patient is a 13-year-old boy who was referred to our pediatric pulmonology clinic for an occasional “whistling cough” exacerbated by exercise and viral respiratory infections, which was not responsive to bronchodilators or inhaled steroids.

His past medical history was significant for being born prematurely at 29 weeks gestational age and requiring prolonged stay (three months) in the neonatal intensive care unit (NICU) due to respiratory distress syndrome. Early chest X-rays (CXRs) reported changes consistent with bronchopulmonary dysplasia (BPD) and bilateral pulmonary interstitial emphysema (PIE). At 19 days of life, the patient developed complete right lung collapse, which was not responsive to suctioning and chest physiotherapy. Bronchoscopy was performed three times (one flexible and two rigid) during his extended NICU stay. The initial rigid bronchoscopy findings revealed a small membrane in the right main stem bronchus but was not mentioned on the two subsequent flexible bronchoscopy reports. All three bronchoscopies reported excessive airway sections.

The patient was discharged home from NICU on room air but with persistent right middle lobe (RML) and right lower lobe (RLL) collapse on chest X-ray. During the first five years of life, the patient had recurrent chest infections requiring short hospitalizations and antibiotics administration. Subsequently, the frequency of chest infections decreased with time until he was referred to us at the age of 13 years because of the whistling cough.

By our evaluation, patient appeared healthy except for mild scoliosis. Vital signs were normal. Oxygen saturation was 96% on room air. Chest examination was remarkable for decreased air entry, dullness, and monophonic wheezing over the right hemithorax. Cardiovascular examination was significant for right-displacement of heart sounds with no murmurs.

**Fig. 1:**
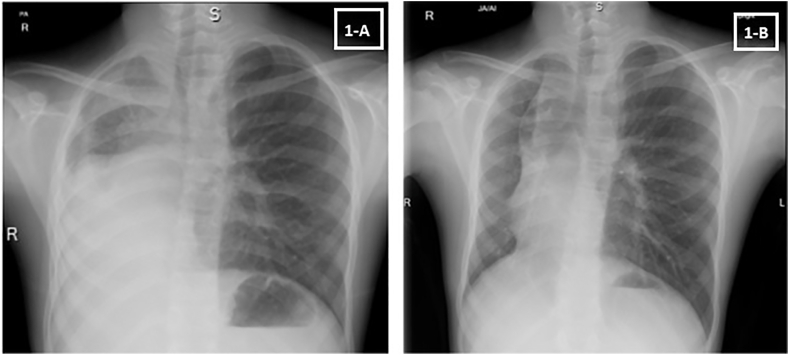
(A) Chest X-ray at presentation (B) Chest X-ray after ablation.

**Fig. 2:**
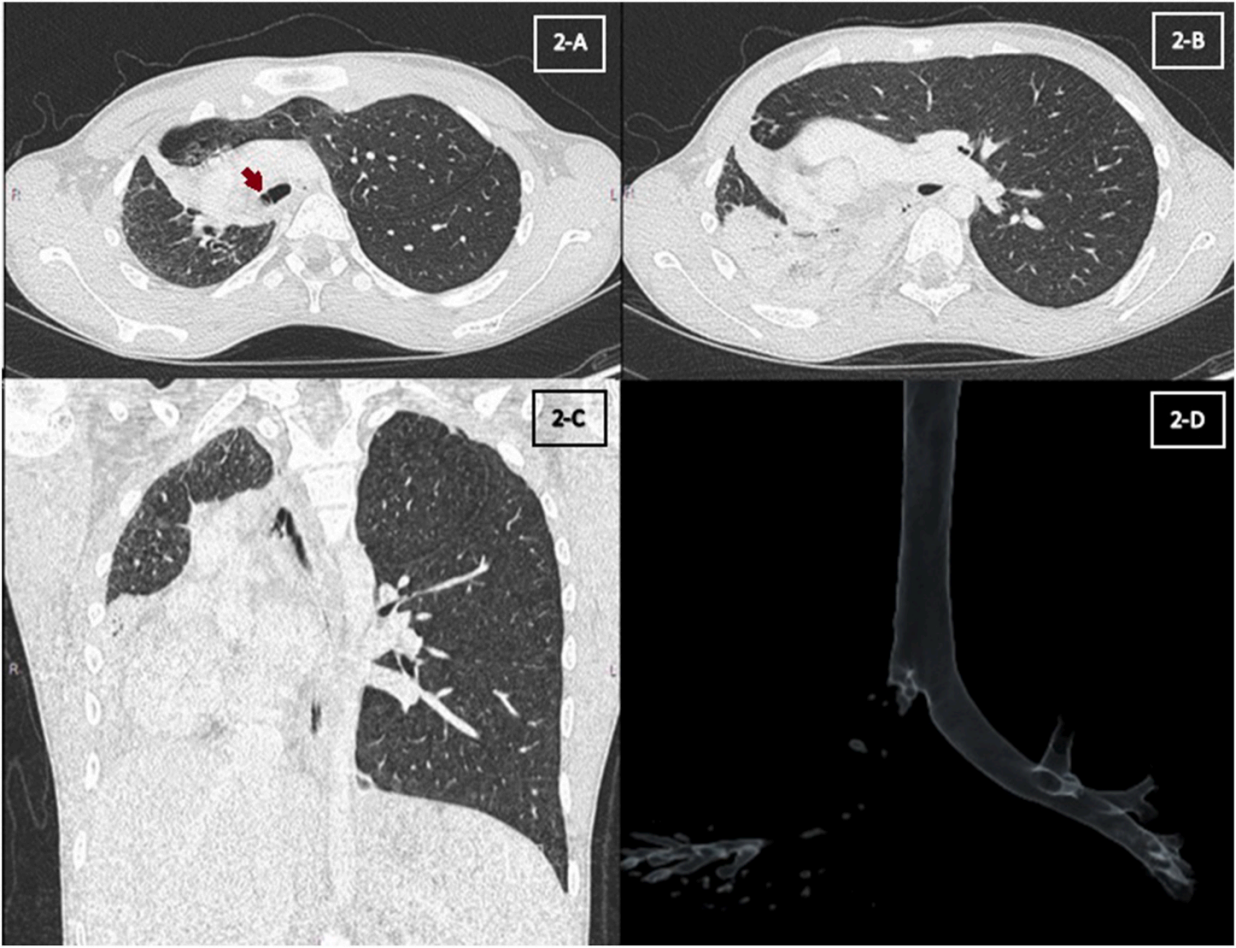
(A) Chest Computed Tomography - Transverse section(Red arrow shows bronchial web) (B) Chest Computed Tomography - Transverse section showing right lobar collapse (C) Chest Computed Tomography - Coronal section (D) Chest Computed Tomography - Three-dimensional image of the airways. (For interpretation of the references to colour in this figure legend, the reader is referred to the web version of this article.)

**Fig. 3:**
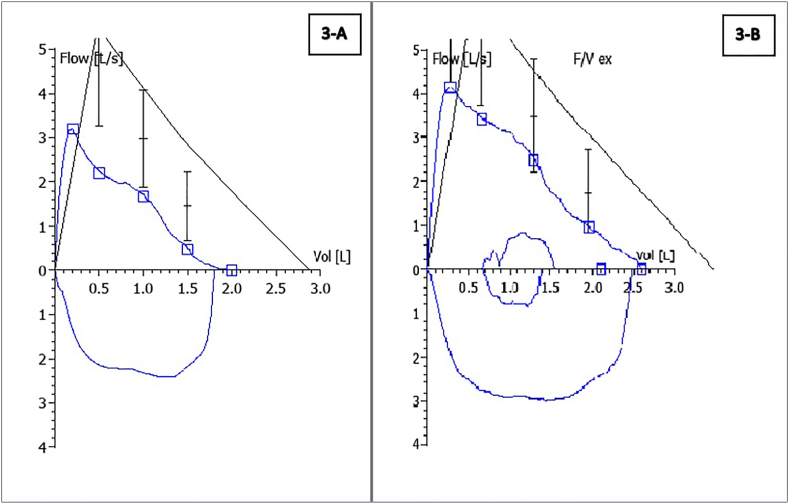
(A) Spirometry at presentation (B) Spirometry after one-month follow-up post ablation.

Chest X-ray showed an overall small right lung with persistent RML and RLL atelectasis and mediastinal shift to the right (Fig. 1-A). Chest computed tomography (CT) with contrast showed atelectasis and traction bronchiectasis involving RML and RLL with hypoplastic right pulmonary artery (RPA) (Fig. 2-A, B, C and D). Echocardiography showed dextro-cardiac position and severely hypoplastic RPA with the absent flow in right pulmonary veins. Spirometry showed flattening of the expiratory phase of the flow volume loop consistent with intrathoracic large airway obstruction pattern (Fig. 3-A). Mild air trapping was noted by body plethysmography.

**Fig. 4:**
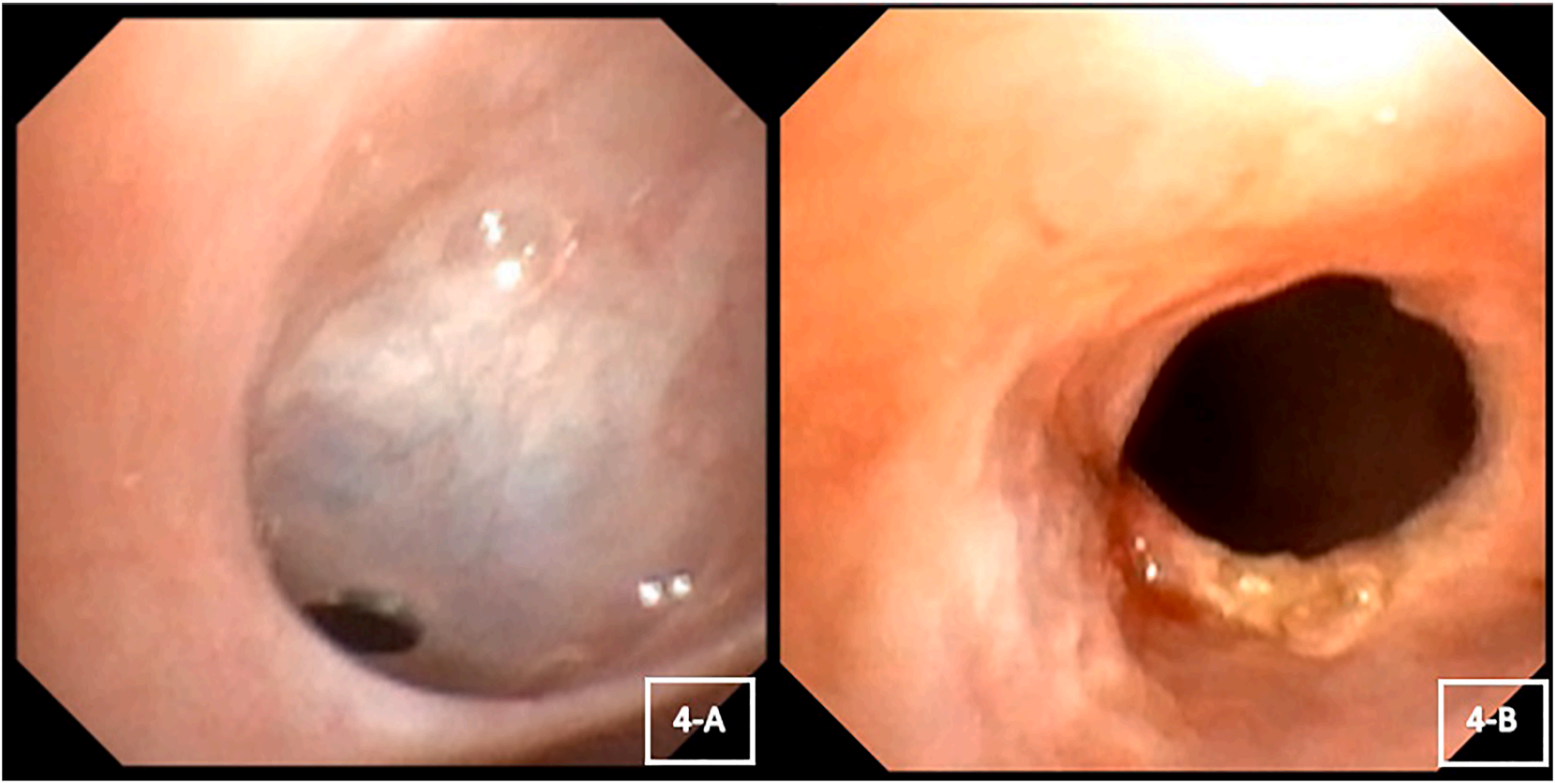
(A) Flexible bronchoscopy view showing the bronchial web at the take-off of right main-stem bronchus (B) Flexible bronchoscopy view post ablation and dilation of the bronchial web.

Diagnostic flexible bronchoscopy was then performed, which revealed a thin fibrovascular membrane occluding the lumen of the right main bronchus except for a tiny opening (about 2 mm) at its periphery (Fig. 4-A). Secretions were noted at the top of the membrane. An attempt to pass a 2.8 mm flexible scope through the small opening was unsuccessful. No attempt was made to treat the web during the procedure.

Treatment of the bronchial web with ablation using argon plasma coagulation (APC) and balloon dilatation was discussed at a multidisciplinary meeting. The goal of the procedure was to relieve airway obstruction, mobilize secretions, and improve exercise endurance, if possible, even though expansion of the chronically collapsed lung was in doubt.

The patient underwent the APC procedure followed by balloon dilatation under general anesthesia and mechanical ventilation using a flexible bronchoscope via an endotracheal tube (ETT). APC was applied in short pulses with an application time of 1–2 seconds each, during which oxygen was discontinued to avoid airway fire. The interval between pulses was around 2–3 seconds. Pulses were applied first around the opening then moving towards the periphery. APC settings used were: power of 20 W and gas flow rate of 0.5 L/min. After APC ablation of most of the web was achieved, repeated balloon dilatation was performed using 6 mm balloons that were inflated for 30 seconds twice.

APC ablation and dilatation resulted in near-complete resolution of the obstruction with a remnant edge (Fig. 4-B). After ablation, the scope was advanced beyond the right main stem bronchus, the right upper lobe bronchus, and the bronchus intermedius, which appeared patent but contained small amounts of clear thick secretion. Mild malacia of RML and RLL bronchi were also noted.

The patient tolerated the procedure with no blood loss and he was discharged home on the same day on room air. A month after the procedure, the patient reported easier breathing, better appetite, and exercise tolerance during a follow-up clinic visit. The previously reported whistling cough has completely disappeared.

**Fig. 5:**
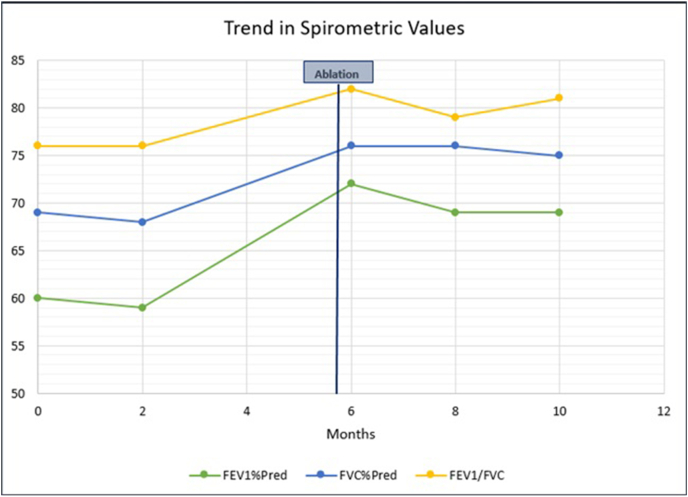
Trend in spirometric values prior and post ablation.

Post-procedure spirometry revealed significant improvement in forced expiratory volume in 1 second (FEV1%) by 540 ml and forced vital capacity (FVC) by 520 ml compared to pre-procedure spirometry (Fig. 3-B and Fig. 5). Additionally, no more air trapping was noted by body plethysmography. Chest x-ray showed re-expansion of the right lower and middle lobes, but the fully expanded right lung continued to appear small (hypoplastic) compared to the left lung (Fig. 1-B). Repeat echocardiography showed improved RPA size from 4 mm to 7.5 mm, indicating mild hypoplasia with improved blood flow.

The patient continues to follow in our pulmonology clinic closely to observe for signs of reformation of the web or recurrence of lung collapse (re-emergence of symptoms or decline in FEV1%), which might require re-evaluation with flexible bronchoscopy.

## 2Discussion

A Bronchial web is a congenital or acquired airway abnormality that can cause significant airway obstruction. Most acquired cases of bronchial webs are secondary to trauma, infections, or post-lung transplant. Congenital bronchial webs, on the other hand, are extremely rare. Table 1 summarizes all published cases of acquired and congenital bronchial webs, including their clinical presentation, treatments, and clinical outcomes [[1], [2], [3], [4], [5], [6], [7], [8], [9], [10], [11]].

**Table 1:** Published cases of acquired and congenital bronchial webs with clinical presentation, treatments, and clinical outcomes.

Report	Number of cases	Etiology	Age at diagnosis	Presenting Symptoms	Management	Outcome
Kompf et al., 1974 [1]	1	Congenital	Newborn	Respiratory obstruction (Distress)	Trans bronchial incision	Curative
Patronas et al., 1976 [2]	1	Congenital	47 years	Persistent “bronchitis” Hemoptysis	None (Biopsied)	Not reported
Udwadia et al., 1990 [3]	2	Acquired (Sarcoidosis)	Adults	Unspecified	Unspecified	Unspecified
Takayama et al., 1991 [4]	1	Congenital	18 years	Intermittent hemoptysis	Broken by biopsy	Not reported
Kovitz et al., 2002 [5]	1	Acquired (Trauma)	71 years	Dyspnea Cough	Puncture APC Balloon Stent	Improved
Bugmann et al., 2003 [6]	1	Acquired (Post FBA)	3 years	Recurrent Bronchitis 4 months after FBA removal	Biopsy Forceps	Resolution of cough
Colin et al., 2006 [7]	3	Acquired (CF) 1 CF after transplant	1: 26 years 2: 26 years 3: 37 years	1: Chest pain, SOB 2: Chest pain 3: Unspecified	1: Unsuccessful endoscopic penetration 2: Transbronchial biopsy forceps, balloon dilatation 3: Transbronchial biopsy forceps, balloon dilatation	1: Unknown 2: Pneumothorax and recurrence 3: Improved
Keating et al., 2011 [8]	1	Acquired (Post transplant)	58 years	Dyspnea Productive cough	APC and Balloon dilatation (Multiple)	Recurrence Distal extension
Medrek et al., 2016 [9]	1	Acquired (Influenza)	35 years	Respiratory failure post influenza Bronchiectasis	None	Death
Crowhurst et al., 2019 [10]	1	Acquired (Chemical inhalation)	47 years	Dyspnea Productive cough	Wang Puncture Balloon dilatation	Recurrence Improved post lung transplant
Hashim et al., 2019 [11]	1	Acquired (Cryglobinemia)	68 years	Hypoxemia Refractory pneumonia	Steroids Transbronchial biopsies	Improved hypoxia

Bronchial webs can result in atelectasis or hyperinflation due to complete or partial airway obstruction. Bronchial webs are not easily differentiated from other cases of bronchial obstruction (i.e., foreign body aspiration, tumors, endobronchial TB, severe airway malacia, or atresia) based on clinical and radiological presentation alone. Flexible bronchoscopy is the diagnostic procedure of choice for all causes of bronchial obstruction and, therefore, is indicated in all cases of unexplained persistent unilateral atelectasis or hyperinflation.

If a diagnosis of the bronchial web is missed or left untreated, it can lead to bronchiectasis and/or permanent lung damage, hypoxemia, hypoventilation, and/or exercise limitation [10]. Fortunately, the lifelong right lower lobe collapse that our patient had was reversed by treating the web and opening the right main stem. However, the entire right lung remained smaller than the left lung, and the right main pulmonary artery was small despite the improved blood flow post-procedure. This highlights the importance of early detection and treatment of bronchial webs.

Definitive treatment of bronchial webs can be achieved by surgical removal of the web using various techniques, which include open and endoscopic procedures. Removal of the web using flexible bronchoscopy is the safest approach. Different endoscopic methods have been reported, including Wang needle, Laser, APC, balloon dilatation, or various combinations of these techniques [5]. In our patient, APC ablation followed by balloon dilatation of the congenital web was safe. It resulted in a complete opening of the bronchus with no evidence of recurrence six months later. Recurrence has been reported predominantly in acquired bronchial webs [5]. Therefore, a repeated procedure may be required. Airway stents can also be used for the prevention of recurrence and the development of airway stenosis. Despite the developing knowledge and experience of airway stenting in children, they may cause severe complications such as stent migration, granulation or even serious bleeding and airways erosion [12].

In conclusion, our case represents a rare cause of airway obstruction. Our case highlights the clinical knowledge and the bronchoscopic intervention that are rarely encountered in pediatric age group. Moreover, it demonstrates how early recognition and intervention diagnosis lead to favorable clinical outcomes.

## Declaration of competing interest

None.

Non-funded article.
